# Longitudinal Correlation of Frequency-to-Place Mismatch and Postoperative Speech Perception Outcomes in Cochlear Implant Recipients: Monosyllable, Consonant, Word, and Sentence

**DOI:** 10.3390/audiolres16020056

**Published:** 2026-04-10

**Authors:** Toshihito Sahara, Yujiro Hoshi, Anjin Mori, Hajime Koyama, Yasuhiro Osaki, Waki Nakajima, Takeshi Fujita, Akinori Kashio, Katsumi Doi

**Affiliations:** 1 Department of Otolaryngology and Head and Neck Surgery, Faculty of Medicine, University of Tokyo, Tokyo 113-8655, Japan; sahatarian@yahoo.co.jp (T.S.); a.h.cis.d.e.fis.gis.a@gmail.com (A.M.); harufsf@gmail.com (H.K.); kashioa@gmail.com (A.K.); 2 Department of Otorhinolaryngology, Faculty of Medicine, Kindai University, Higashiosaka 589-8511, Japan; 3 Department of Physiology, Graduate School of Medicine, Yokohama City University, Yokohama 236-0004, Japan; waki@yokohama-cu.ac.jp; 4 Department of Otorhinolaryngology, Mitsui Memorial Hospital, Tokyo 101-8643, Japan; 5 Department of Otorhinolaryngology, Ikeda City Hospital, Ikeda 563-8510, Japan; aac61130@pop02.odn.ne.jp; 6 Department of Otolaryngology—Head and Neck Surgery, Graduate School of Medicine, Kobe University, Kobe 650-0017, Japan; 7 Ear Center, Iseikai International General Hospital, Osaka 530-0052, Japan

**Keywords:** cochlear implant, frequency-to-place mismatch, speech perception outcomes, cochlear duct length, OTOPLAN, CI-2004, auditory plasticity

## Abstract

**Background/Objectives**: Frequency-to-place mismatch between cochlear implant (CI) electrodes and cochlear tonotopy has been suggested to affect postoperative speech perception. This study aimed to examine the associations between frequency-to-place mismatch and speech perception outcomes across multiple linguistic levels in patients with CI and to assess how these associations change over time using postoperative computed tomography. **Methods**: This retrospective cohort study included 44 postlingually deafened adults who underwent unilateral cochlear implantation with a Flex28 electrode by a single surgeon at a tertiary care hospital. Speech perception was assessed using CI-2004, a Japanese speech perception test consisting of monosyllables, consonants, words, and sentences, in quiet settings at 3, 6, and 12 months after CI activation. Partial correlation analyses between frequency-to-place mismatch and postoperative speech perception scores were performed in 35 of the 44 patients, controlling for age and mean preoperative pure-tone thresholds. **Results**: Negative associations were observed between frequency-to-place mismatch and CI-2004 scores, particularly for monosyllable and consonant perception in uncorrected analyses. After correction for multiple comparisons, only consonant perception at 3 months after CI activation remained significant (r = −0.52, *p* = 0.002). Similar patterns were observed for other speech measures and at later time points, although these did not remain significant after correction. **Conclusions**: Frequency-to-place mismatch was associated with postoperative speech perception outcomes, particularly those involving phoneme-level recognition. After correction for multiple comparisons, only consonant perception at 3 months after CI activation remained significant.

## 1. Introduction

The human cochlea has a spiral structure with approximately 2½ turns. The mapping frequency to each cochlear position creates a tonotopic array structure that fits an exponential function, with the lowest frequencies at the apical turn and the highest frequencies at the basal turn [1,2]. This tonotopic array structure is important for auditory mechanisms. The cochlear size and shape without malformations differ between individuals [3,4,5], and one of the most fundamental cochlear size parameters is the cochlear duct length (CDL), which is related to the tonotopic array structure. The study of CDL measurement in histological specimens began in the 19th century, and with the advancement of computed tomography (CT), various methods of measuring CDL have been reported [3,6,7,8,9], including the Escudé equation, which uses the diameter of the cochlear basal turn [10], and the Elliptic–Circular Approximation equation, which uses both the width and diameter of the cochlear basal turn [11,12]. The CDL has been reported to range from approximately 30 to 37 mm in most contemporary CT-based studies [3,4,7,9,13], although historical reports have described a wider range of 24–45 mm depending on the measurement method [8]. The angular insertion depth (AID) of the apical electrode of a cochlear implant (CI) differs between individuals because of anatomical variations in cochlear size, including the CDL, even when the same type of CI is fully inserted into the cochlea [14,15]. Different AIDs change the tonotopic frequency of the spiral ganglion stimulated by each electrode. However, each electrode uses a default frequency filter. Therefore, the discrepancy between these frequencies is called frequency-to-place mismatch [16,17,18,19]. Frequency-to-place mismatch can be a problem, particularly in post-lingually deafened patients, owing to the discrepancy between the innate tonotopic frequency and the default mapping frequency of the CI. Therefore, an accurate prediction of the frequency of position mapping using a tonotopic chart within the cochlea is important in cochlear measurements.

A negative correlation between frequency-to-place mismatch and speech perception outcomes of CI has been previously reported. While patients with CI may adapt to the shifted mapping frequency to some extent [20,21,22], this frequency adaptation can be incomplete if the frequency-to-place mismatch is too large because of the inappropriate positioning of the electrodes [23,24]. Some studies have suggested that greater frequency-to-place mismatch is associated with poorer speech perception and delayed postoperative improvement [17,19,25,26,27], whereas other studies have shown no significant effect of frequency-to-place mismatch on speech perception performance [28,29]. Thus, the influence of the frequency-to-place mismatch on speech perception remains unclear and has not been investigated from a longitudinal perspective. Recently developed software can estimate the CDL, AID, electrode position, and frequency corresponding to each electrode, including frequency-to-place mismatch, by pointing to multiple landmarks in CT images [14]. Using this software with each postoperative CT scan, we aimed to examine the associations between frequency-to-place mismatch and speech perception outcomes across multiple linguistic levels in patients with CI, and to assess how these associations change over time using postoperative computed tomography.

## 2. Materials and Methods

### 2.1. Participants

**Figure 1:**
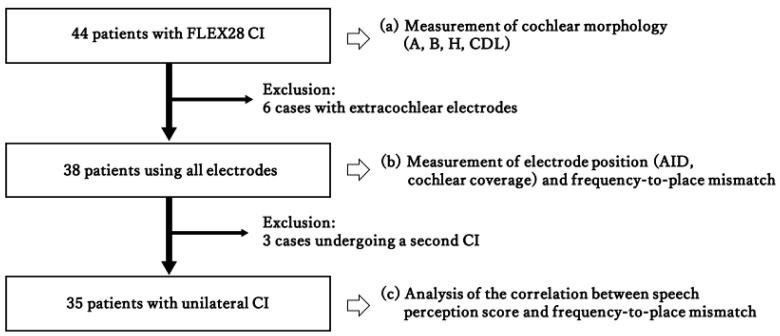
Inclusion criteria. The exclusion criteria are as follows: presence of extracochlear electrodes and a second cochlear implantation.

This retrospective study was conducted in accordance with the Declaration of Helsinki (1975, revised in 2013) and was approved by the local ethics committee (Approval No. R02-086). Written informed consent for participation in the study and use of clinical data was obtained from all participants. According to the study protocol, patients who underwent cochlear implantation at our institution between January 2012 and December 2021 were retrospectively screened. The final study cohort consisted of 44 native Japanese speakers who received a Flex28 electrode (MED-EL, Innsbruck, Austria) between March 2014 and June 2020, including 15 males and 29 females, aged 21–89 years (mean: 67.0 ± 14.2 years). Children under 18 years of age, those with cochlear malformations, and those without proper images for analysis in a sufficiently thin slice setting with a thickness of ≤0.625 mm were excluded from this study. All patients were postlingually deaf. Cochlear implantations were performed by the same surgeon (K.D.) using the round window approach (minimal round window membrane opening, without the extended round window approach or cochleostomy, topical dexamethasone at the insertion site, and slow insertion of approximately 2 min) to reduce cochlear trauma, optimize neural viability, and preserve any residual hearing. The electrodes were inserted up to the mark according to the manufacturer’s instructions, but six cases resulted in partial insertion owing to resistance in the middle. No shorter electrode array was used in this study. All patients received the same Flex28 electrode, and reduced cochlear coverage in some cases reflected partial insertion rather than the use of a shorter array. Default frequency filters for CI-alone device users were obtained using the manufacturer’s software. The frequency range was 70–8500 Hz and was logarithmically distributed across the active electrodes. Preoperative image analysis was performed in all 44 patients implanted with Flex28 electrodes, which are 28 mm straight lateral wall electrodes with 12 contacts. Cochlear morphology, including the CDL, was measured by the same rater. Electrode position analysis was performed in 38 patients, excluding six patients with extracochlear electrodes. The correlations between the CDL and AID and between CDL and frequency-to-place mismatch were examined in these patients. Three patients were excluded from the analysis of speech perception because they already had a contralateral CI and were undergoing a second CI surgery. Thirty-five patients had unilateral CIs. They all used the FS4 or FS4p coding strategy with default center frequencies, and no electrodes were deactivated in any subject. Programming was performed by two experienced speech-language-hearing therapists at our institution according to routine clinical practice. Detailed information regarding all individual fitting adjustments during follow-up was not available. For the hearing outcome analysis, the correlation between speech perception scores and frequency-to-place mismatches was analyzed. A summary of the eligibility criteria is shown in Figure 1. The demographic and clinical characteristics of the 35 patients included in the frequency-to-place mismatch analysis are summarized in Table 1.

### 2.2. Image Analysis and Software

**Figure 2:**
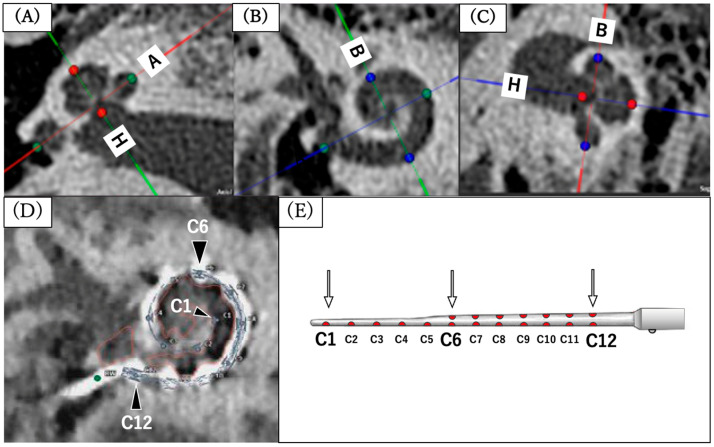
Measurement of cochlear parameters and electrode localization using CT-based analysis (OTOPLAN). (**A**–**C**) Measurement of cochlear basal turn diameter (A), width (B), and cochlear height (H). (**D**) Electrode localization and angular insertion depth. (**E**) FLEX28 electrode array and electrode numbering.

Image analysis was performed using OTOPLAN software (version 3.0; MED-EL and CAScination AG, Bern, Switzerland). The CT data were uploaded to the software in digital imaging and communications in medicine file format, and the data passed an integrity check for missing slices and consistency of distances between slices before analysis. The “A value” (the basal turn diameter; the largest distance from the center of the round window to the contralateral wall) and “B value” (the basal turn width; the distance between the cochlear walls perpendicular to the A value line) were measured on an image plane parallel to the basal turn of the cochlea. “H value” (the cochlear height; the distance between the cochlear base and the apex) was measured on an orthogonal plane (Figure 2A–C). Each value was measured using the “cochlear view,” which displays the three-dimensional, cylindrical structure of the cochlea, with the modiolus chosen as the *z*-axis [30]. The CDL was calculated based on the measured A and B values using the elliptic–circular approximation equation [11,12]. Data analysis using multiplanar reformation was performed by a single rater experienced in the use of this software. The AID was defined as the angular position of the apical electrode (C1) contact (Figure 2D) [14,30,31]. The zero-reference angle was selected at the center of the round window, which is closely related to the basal end of the organ of Corti. Electrode coverage was calculated by dividing the length of the insertion electrode by the CDL. The correlation between CDL and AID was also evaluated. The frequency corresponding to each electrode location was approximated according to the Greenwood frequency equation [2] at the level of the organ of Corti. The electrode contacts were numbered from the apex (C1) to the base (C12) according to the manufacturer’s convention (Figure 2E).

### 2.3. Quantification of Frequency-to-Place Mismatch

In postoperative mapping, default frequencies were assigned to all patients with CI, resulting in frequency-to-place mismatches owing to variations in the electrode insertion depth. To quantify the extent of frequency-to-place mismatch, a fourth-order polynomial function was fit to the semitone deviation between the assigned center frequency and the Greenwood-predicted frequency as a function of AID for each ear [32]. As previously reported [19], absolute semitone deviation was quantified in the tonotopic region corresponding to approximately 1–2 kHz, which represents the spectral center of speech information relevant for recognition. For statistical analysis, frequency-to-place mismatch at this region was evaluated at a predefined electrode (C6) to allow consistent comparison across subjects. C6 was selected because it corresponds to the mid-frequency region (1–2 kHz), which is critical for speech recognition. This approach prioritizes consistency across subjects and targets the frequency region most relevant for speech perception.

### 2.4. Postoperative Speech Perception Evaluation

Speech perception was evaluated using CI-2004 (the Japanese speech perception test consisting of monosyllables, consonants, words, and sentences in quiet; 70 dB SPL) [33,34]. Testing was performed in a soundproof room with the patient seated 1 m from the sound source under unilateral CI listening conditions using the default frequency allocation and without using a hearing aid in the contralateral ear at 3, 6, and 12 months after CI activation. During the examination, an earmuff was used in the contralateral ear. Performance was expressed as the percentage of correctly identified items, ranging from 0% to 100%. In the CI-2004 battery, the consonant and monosyllable subtests primarily assess perception at the phoneme or syllable level with minimal lexical–semantic support. In particular, the consonant subtest evaluates consonant identification using stimuli in which the following vowel is fixed to /a/. By contrast, the word and sentence subtests assess perception of meaningful speech materials. In the word subtest, each item is scored as correct or incorrect at the whole-word level, rather than by awarding partial credit for individual phonemes within a word, as in the CNC test. In the sentence subtest, scoring is based on correctly identified smaller speech units within each sentence rather than on all-or-none sentence-level accuracy.

### 2.5. Statistical Analysis

Data management was conducted using Microsoft Excel (version 16.75.2 for iOS; Microsoft Corporation, Redmond, WA, USA). Statistical analysis was performed using JMP Pro (version 17.2.0; SAS Institute Inc., Cary, NC, USA) and SPSS Statistics (version 29.0.0.0; IBM Corp., Armonk, NY, USA). The *t*-test was used to evaluate the cochlear parameters between sex and side. Two-sided Pearson correlation coefficients were applied to evaluate the correlation between the AID and CDL, frequency-to-place mismatch, and various values, such as the AID, electrode coverage, and CDL. To examine the relationship between frequency-to-place mismatch and speech perception scores while controlling for potential confounding factors, we conducted partial correlation analyses. Age was included as a covariate based on prior evidence of its influence on speech perception outcomes. Preoperative mean air-conduction thresholds at 500, 1000, and 2000 Hz were also considered as a potential confounding variable and showed significant correlations with some of the speech perception scores. Therefore, both age and preoperative audiometric data were included as control variables in the partial correlation analyses. Other potential confounders, such as duration of hearing loss and daily device usage, were not available in this dataset. Statistical significance was set at *p* < 0.05. To account for multiple comparisons in the partial correlation analyses, Bonferroni correction was additionally applied. Both uncorrected and corrected results are reported, and findings that did not remain significant after correction were interpreted cautiously.

## 3. Results

### 3.1. Cochlear Morphological Parameters and Electrode Position

**Figure 3:**
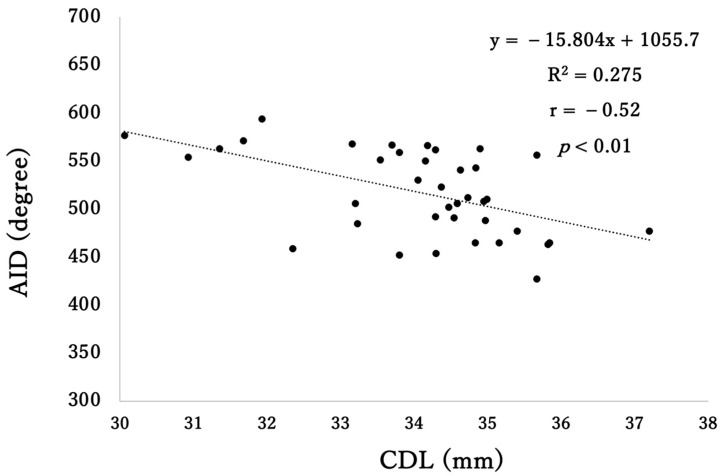
Correlation between cochlear duct length (CDL) and angular insertion depth (AID) in Flex28 (n = 38). CDL, cochlear duct length; AID, angular insertion depth.

The mean A, B, H values, and CDL were 8.7 ± 0.4 mm, 6.5 ± 0.3 mm, 4.0 ± 0.3 mm, and 34.0 ± 1.4 mm (range: 30.1–35.8 mm), respectively, in 44 patients. No significant difference was observed between the left and right sides of the CDL (right: 34.2 ± 1.3 mm, left: 33.5 ± 1.5 mm; *p* = 0.10). In contrast, a significant difference was observed in the CDL between men and women, with 34.6 ± 0.9 mm for men and 33.6 ± 1.5 mm for women (*p* < 0.05). In 38 patients (86.4%), all electrodes were fully inserted and included in the analysis. No cases were observed with apical electrode insertion beyond 720°. In this subgroup, the mean AID was 511.1 ± 51.2 degrees, the electrode coverage was 74.8 ± 4.6%, and the estimated frequency of the apical electrode was 286.4 ± 119.9 Hz (Table 2). Although the electrodes were inserted into the scala tympani along the lateral wall and their positions were somewhat variable, there was a significant negative correlation between the AID and CDL (r = −0.52, *p* < 0.01), indicating that longer CDL was associated with smaller AID (Figure 3).

### 3.2. Analysis of Frequency-to-Place Mismatch

Most cases had a frequency-to-place mismatch owing to shallow electrodes, and there were no cases with mismatch owing to deeply inserted electrodes.

**Figure 4:**
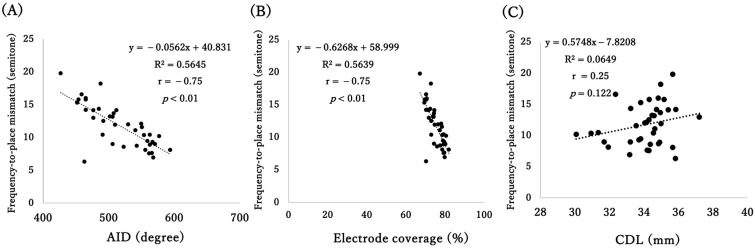
Correlation between frequency-to-place mismatch (semitone) and the angular insertion depth (**A**), electrode coverage (**B**), and cochlear duct length (**C**) (n = 38).

Each insertion electrode was assigned a default frequency map; therefore, a larger frequency-to-place mismatch was expected if the angle of the C6 was shallower than that of the originally designed condition. This led to a negative correlation between the AID and frequency-to-place mismatch (r = −0.75, *p* < 0.01) (Figure 4A) and also between electrode coverage and frequency-to-place mismatch (r = −0.75, *p* < 0.01) (Figure 4B). No correlation was observed between the CDL and frequency-to-place mismatch (r = 0.25, *p* = 0.122) (Figure 4C).

### 3.3. Partial Correlation of Frequency-to-Place Mismatch with Speech Perception Outcomes

At 3, 6, and 12 months after CI activation, the partial correlation coefficients between CI-2004 score and frequency-to-place mismatch using age and the mean preoperative air-conduction threshold as confounding factors were as follows: monosyllables, −0.41 (*p* = 0.019), −0.41 (*p* = 0.022), −0.40 (*p* = 0.038); consonants, −0.52 (*p* = 0.002), −0.41 (*p* = 0.024), −0.50 (*p* = 0.008); words, −0.39 (*p* = 0.023), −0.39 (*p* = 0.032), −0.20 (*p* = 0.316); and sentences, −0.35 (*p* = 0.045), −0.28 (*p* = 0.132), −0.13 (*p* = 0.514) (Table 3).

Owing to some missing data, 35, 33, and 27 patients were included in the group at 3, 6, and 12 months after CI activation, respectively. In the uncorrected analyses, negative associations were observed between frequency-to-place mismatch and several speech perception measures, with the strongest association observed for consonant perception at 3 months (r = −0.52). This was the only association that remained significant after correction for multiple comparisons. Similar patterns were observed for monosyllable and consonant perception at later time points, although these did not remain significant after correction. Correlations between frequency-to-place mismatch and speech perception outcomes without adjustment for covariates are shown in Supplementary Figure S1. Additional partial correlation analyses were performed to examine the relationships between speech perception outcomes and both CDL and AID, using the same covariates. No meaningful correlations were observed between CDL and speech perception outcomes at any time point. For AID, several associations were observed in the uncorrected analyses, particularly for consonant perception and some phoneme-level measures. However, none remained significant after multiple comparisons (Supplementary Tables S1 and S2).

Partial correlation analyses were performed on frequency-to-place mismatch (semitone) and speech perception outcomes (CI-2004), controlling for age and mean preoperative air-conduction (500, 1000, 2000 Hz). Gray background indicates statistical significance after Bonferroni correction for multiple comparisons (corrected significance threshold: *p* < 0.0042). Data in separate columns indicate the results at 3 months (n = 35), 6 months (n = 33), and 12 months (n = 27) after activation for unilateral cochlear implant users.

## 4. Discussion

The mean A, B, and H values in this study were consistent with previous reports [3,7,13,15], as was the mean CDL [4,9,13]. While no significant difference was observed in CDL laterality, male participants showed significantly longer CDL than female participants. This finding is consistent with previous studies reporting sex-related differences in cochlear size and CDL [4,15,34]. A strong negative correlation was observed between the frequency-to-place mismatch and AID or electrode coverage, indicating that deeper electrode insertion was associated with smaller mismatch. Considering the negative correlation between the CDL and AID, one might expect that a larger CDL would be associated with a shallower AID and, consequently, a greater frequency-to-place mismatch when the same type of electrode is inserted, and default mapping is performed. However, CDL was not directly correlated with frequency-to-place mismatch, suggesting that cochlear size alone does not determine mismatch under default mapping. In addition, actual insertion depth may vary even with the same electrode model and nominal insertion mark, depending on the intracochlear course of the electrode within the scala tympani. As previously reported, deeper insertion not only allows for reduced frequency-to-place mismatch but also greater angular separation between adjacent electrodes, which theoretically may facilitate more discrete stimulation of neuronal populations despite the spread of excitation [19]. Deeper insertion of electrodes in straight arrays has been associated with better speech perception outcomes in several studies [35,36,37]. However, in this study, it was difficult to distinguish whether the observed associations with speech perception were more closely related to deeper electrode insertion or to smaller frequency-to-place mismatch, because deeper insertion was generally associated with smaller mismatch. In additional analyses, AID showed several associations in the uncorrected analyses, especially for phoneme-level measures, but none remained significant after correction for multiple comparisons. CDL itself was not correlated with speech perception outcomes at any time point.

After correction for multiple comparisons, only the correlation between frequency-to-place mismatch and consonant perception at 3 months remained statistically significant. This suggests that the association with frequency-to-place mismatch may be particularly relevant to early postoperative phoneme-level perception compared with the other speech measures examined. Canfarotta et al. examined the frequency-to-place mismatch and speech perception of CI recipients using the Consonant-Nucleus-Consonant (CNC) word test at 1, 3, and 6 months after implant activation and found a significant negative correlation [19]. These findings are partially consistent with the present results. However, the CI-2004 monosyllable and consonant subtests differ somewhat from the CNC word test in that they primarily assess discrimination at the level of a single phoneme or syllable, with minimal lexical–semantic support. In particular, the consonant subtest is designed to evaluate discrimination among consonants presented with the same following vowel, thereby placing greater demands on fine spectral and frequency discrimination. In the present study, consonant perception showed the most robust association with frequency-to-place mismatch, representing a novel aspect of this work. Vowels can be classified into first and second formants [38]. Consonant-formant spectrograms are more diverse than vowel spectrograms, and consonants with the same vowel can be classified according to their second formant transitions because first formants are not much different [39]. The CI-2004 consonant test determines the discrimination of consonants with the same vowel. Therefore, the CI-2004 consonant test requires more accurate frequency discrimination than the monosyllabic test. Although various factors affect the postoperative speech perception outcomes of CIs, consonants may be particularly well suited for evaluating the effects of frequency-to-place mismatch. Regarding words and sentences, negative associations were observed in the uncorrected analyses, with word perception showing associations up to 6 months post-activation, whereas sentence perception showed only a weak association at 3 months. These findings can be explained in terms of linguistic redundancy. Monosyllable and consonant recognition primarily depend on acoustic information such as frequency characteristics. However, word recognition can use semantic and lexical knowledge, while sentence recognition benefits from further contextual and grammatical cues, thus increasing predictability. Consequently, the disadvantage of frequency-to-place mismatch as an acoustic factor may be mitigated by these higher-order linguistic processes [40,41,42]. The correlation for sentence perception was also limited by the ceiling effect, as 45.5% and 63.0% of patients achieved scores above 80% at 6 and 12 months post-activation, respectively.

For monosyllable and consonant perception, negative associations with frequency-to-place mismatch were observed across time points in the uncorrected analysis, with the strongest association observed at the earliest time point of 3 months after activation. A similar temporal pattern was observed for word perception, whereas sentence perception showed only a weak association at 3 months. Several studies have reported on the biological adaptation of the central nervous system to changes in frequency input from the cochlear nerve. Changes in peripheral stimuli reorganize the tonotopic map of the primary auditory cortex and adapt to the new input. In animals, this adaptation can be observed at the level of single-neuron firing, and the earliest reports of reorganization in the primary auditory cortex are hours to days [43,44,45]. This adaptation is not only observed in the central auditory system, including the cerebral cortex, but may also be attributed to changes in the sensitivity of neural and sensory cells in the peripheral cochlear tissue. It has been known that within 3–6 months after cochlear implantation, there is a decrease in impedance and an increase in comfortable stimulation levels [46,47,48]. Furthermore, these changes lead to an expansion of the dynamic range of the CI, which has been associated with improved speech recognition scores [49]. These plastic changes in both central and peripheral systems may, over time, mitigate the effects of mismatch and potentially create differences in the correlation between frequency-to-place mismatch and consonant, word, and sentence perception. This perspective may also help explain why good speech outcomes can still be achieved in recipients of other cochlear implant systems, even when frequency-to-place mismatch or incomplete cochlear coverage is presumed. The present findings suggest that the influence of frequency-to-place mismatch may be more apparent during the early postoperative period. In contrast, its impact may diminish over time as central auditory adaptation and higher-order compensatory mechanisms increasingly contribute to speech perception. In this context, favorable later speech outcomes in other implant systems would not necessarily contradict the importance of frequency-to-place mismatch. Still, they may instead reflect the combined effects of peripheral fitting, rehabilitation, and central adaptation. While the speech perception of the frequency-shifted sound improved over time, a limit of adaptation to frequency shifting was observed [20,32], and the optimal adaptation period of frequency-to-place mismatch remains unclear. The greater the frequency shift, the longer it takes to adjust and the poorer the speech perception is [17]. For monosyllables and consonants, negative associations were also observed at 12 months in the uncorrected analyses, suggesting that adaptation to frequency-to-place mismatch may remain incomplete in some patients over the first postoperative year. Factors influencing speech perception in CI include age, cognitive function, duration of hearing loss and residual hearing level [50,51], and this study showed that the frequency-to-place mismatch could be an additional factor.

This study demonstrated an association between frequency-to-place mismatch and speech perception outcomes. Given the observational design, the findings should be interpreted as associative rather than causal, and residual confounding cannot be excluded. Although evaluating CDL and identifying electrode position using postoperative CT may allow assignment of tonotopically aligned frequencies to individual electrodes, it remains unclear whether such adjustments necessarily lead to improved speech perception outcomes. Notably, Gilbert et al. prospectively evaluated experienced CI users who had long-term exposure to default frequency maps before switching to CT-based allocations and found no group-level benefit [52]. Therefore, while reducing frequency-to-place mismatch may be theoretically advantageous, further prospective and longitudinal studies are warranted to determine its clinical impact.

Study limitations include manual measurements potentially subject to rater error, though previous studies have demonstrated minimal measurement variability [14,53]. This study was exploratory in nature, so findings should be interpreted with caution, particularly given the potential for type I (false-positive) error. Although variables such as duration of deafness, preoperative speech perception, processor generation, and average daily use hours logged are known to be important confounding factors in speech perception outcomes [54], these variables were not included in the present analysis because some data were not consistently available, and preoperative speech perception showed limited contribution. These unmeasured confounders may partially explain the observed associations. Additionally, the electrode type, CI coding strategy, and surgeon were kept consistent. Lastly, the precurved implants with perimodiolar electrodes were excluded from this study because the current software cannot accurately analyze the frequency corresponding to each electrode. In addition, only speech perception was evaluated in this study, and music perception was not assessed. Given that frequency-to-place mismatch may also affect music perception and enjoyment, this remains an important topic for future investigation.

## Figures and Tables

**Table 1:** Demographic and clinical characteristics of the 35 patients in the frequency-to-place mismatch analysis.

Characteristic	Value
Sex	
Male/Female	15/20
Implanted side	
Right/Left	22/13
Age (years)	66.6 ± 16.8
Preoperative hearing level	
Pure-tone average threshold (dB HL) *	97.2 ± 20.2
Maximum speech discrimination score (%) **	29.1 ± 24.1
Etiology of hearing loss	
Unknown/Progressive sensorineural hearing loss	21
Sudden sensorineural hearing loss	2
Chronic otitis media/Cholesteatoma	5
Ménière’s disease/Delayed endolymphatic hydrops	3
Otosclerosis	1
Genetic/Systemic causes ***	3
Internal implant model	
Synchrony	20
Concerto	15

*: Average of the thresholds at 500, 1000, and 2000 Hz. **: Assessed using the Japanese 67-S word list; available for 29 patients. ***: Usher syndrome, Cogan syndrome, and MELAS. Data are presented as mean ± SD unless otherwise indicated.

**Table 2:** Cochlear morphology and electrode position.

Characteristic	Value
Cochlear morphology (n = 44)	
Diameter (A value, mm)	8.7 ± 0.4
Width (B value, mm)	6.5 ± 0.3
Height (H value, mm)	4.0 ± 0.3
Cochlear duct length (CDL, mm)	
Overall	34.0 ± 1.4
Male	34.6 ± 0.9
Female	33.6 ± 1.5
Electrode position (n = 38)	
Angular insertion depth (AID; degrees)	511.1 ± 51.2
Electrode coverage (%)	74.8 ± 4.6
Estimated frequency of the apical electrode (Hz)	286.4 ± 119.9

Data are presented as mean ± SD unless otherwise indicated.

**Table 3:** Partial correlation between frequency-to-place mismatch and speech perception outcomes.

		3 Month		6 Month		12 Month	
		Partial Correlation Coefficient	*p*-Value	Partial Correlation Coefficient	*p*-Value	Partial Correlation Coefficient	*p*-Value
CI-2004	Monosyllable	−0.41	0.019	−0.41	0.022	−0.40	0.038
	Consonant	−0.52	0.002	−0.41	0.024	−0.50	0.008
	Word	−0.39	0.023	−0.39	0.032	−0.20	0.316
	Sentence	−0.35	0.045	−0.28	0.132	−0.13	0.514

## Data Availability

The datasets generated and analyzed during the current study are not publicly available owing to patient confidentiality and ethical restrictions, but they are available from the corresponding author upon reasonable request.

## Supplementary Materials

The following supporting information can be downloaded at: https://www.mdpi.com/article/10.3390/audiolres16020056/s1, Table S1: Partial correlations between cochlear duct length (CDL) and speech perception outcomes. Table S2: Partial correlations between angular insertion depth (AID) and speech perception outcomes. Figure S1: Correlation between frequency-to-place mismatch and speech perception outcomes without confounding factors.

## Abbreviations

The following abbreviations are used in this manuscript:

CI	Cochlear Implant
CT	Computed Tomography
CDL	Cochlear Duct Length
AID	Angular Insertion Depth
