# Supplementary material for: Longitudinal Correlation of Frequency-to-Place Mismatch and Postoperative Speech Perception Outcomes in Cochlear Implant Recipients: Monosyllable, Consonant, Word, and Sentence
Source: Audiol Res. 2026 Apr 10;16(2):56. doi: 10.3390/audiolres16020056 (PMC13114027; doi:10.3390/audiolres16020056)
Supplement: Supplementary file 1 [file audiolres-16-00056-s001.zip › Supplementary Figure S1_AudiolRes.pdf]

Supplementary Figure S1.
Correlation between frequency-to-place mismatch and speech perception outcomes without confounding factors

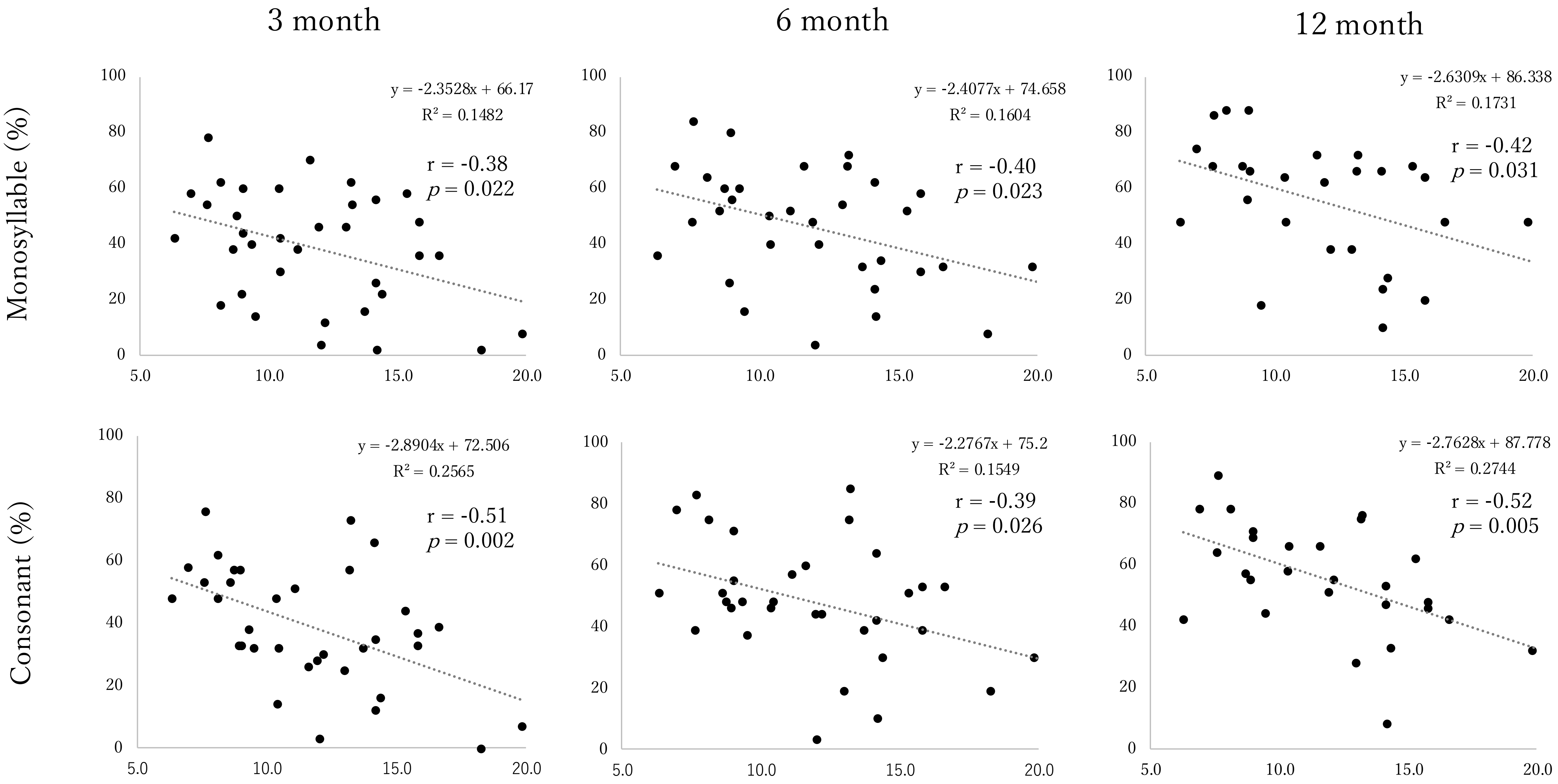

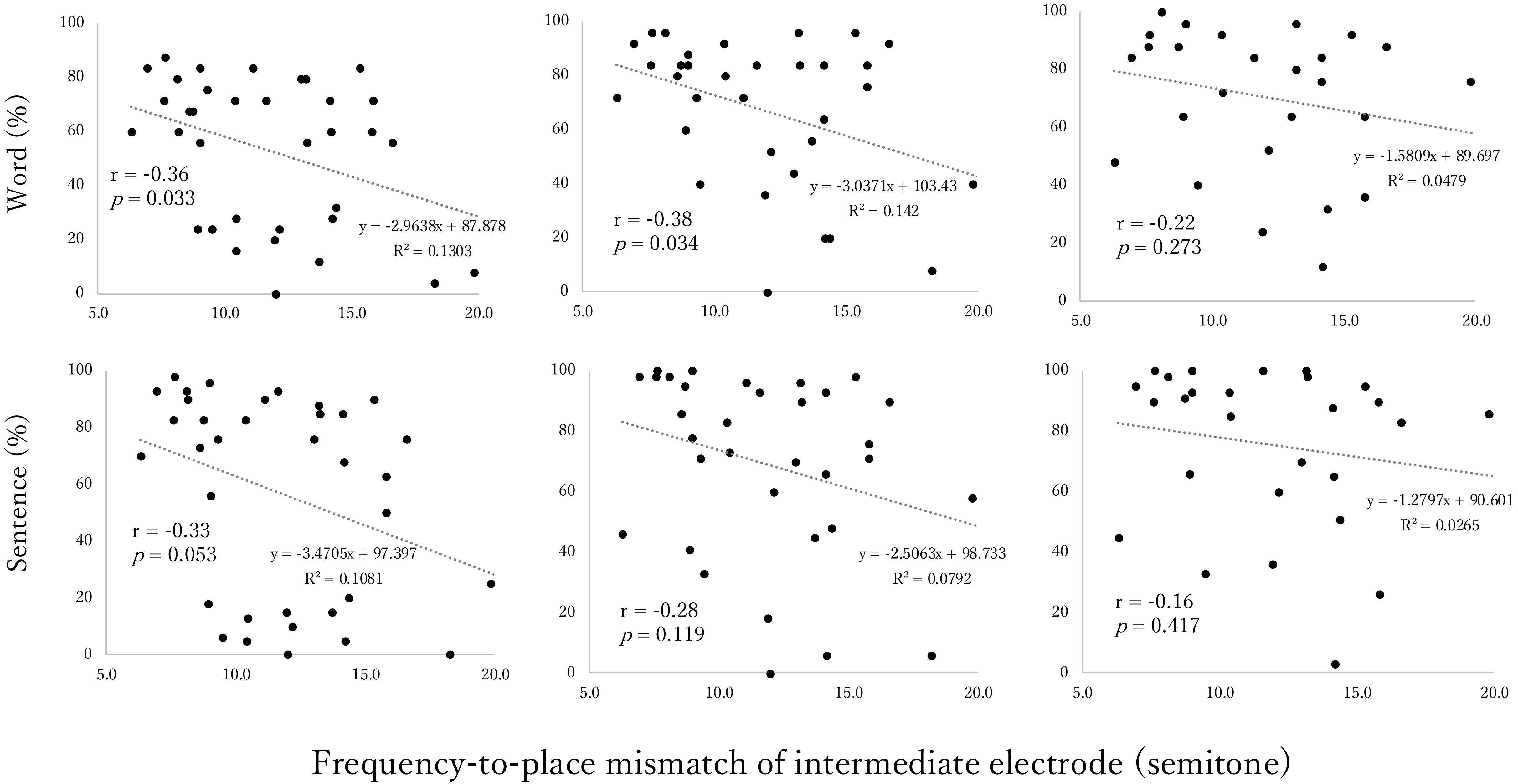
Word (%)
Sentence (%)
r = -0.36
p = 0.033
y = -2.9638x + 87.878
R² = 0.1303
r = -0.38
p = 0.034
y = -3.0371x + 103.43
R² = 0.142
r = -0.22
p = 0.273
y = -1.5809x + 89.697
R² = 0.0479
r = -0.33
p = 0.053
y = -3.4705x + 97.397
R² = 0.1081
r = -0.28
p = 0.119
y = -2.5063x + 98.733
R² = 0.0792
r = -0.16
p = 0.417
y = -1.2797x + 90.601
R² = 0.0265
Frequency-to-place mismatch of intermediate electrode (semitone)
