# Supplementary material for: Longitudinal Correlation of Frequency-to-Place Mismatch and Postoperative Speech Perception Outcomes in Cochlear Implant Recipients: Monosyllable, Consonant, Word, and Sentence
Source: Audiol Res. 2026 Apr 10;16(2):56. doi: 10.3390/audiolres16020056 (PMC13114027; doi:10.3390/audiolres16020056)
Supplement: Supplementary file 1 [file audiolres-16-00056-s001.zip › Supplementary Table S1_AudiolRes.pdf]

Supplementary Table S1.
Partial correlations between cochlear duct length (CDL) and speech perception outcomes

		3 month		6 month		12 month	
		Partial correlation coefficient	*p* -value	Partial correlation coefficient	*p* -value	Partial correlation coefficient	*p* -value
CI-2004	Monosyllable	-0.07	0.708	-0.10	0.610	-0.12	0.562
	Consonant	-0.17	0.340	-0.25	0.184	-0.32	0.118
	Word	-0.07	0.694	-0.33	0.069	-0.29	0.168
	Sentence	-0.16	0.362	-0.23	0.218	-0.21	0.321

Partial correlation analyses were performed, controlling for age and preoperative mean air-conduction thresholds (500, 1000, and 2000 Hz). Bonferroni-corrected significance threshold: $p < 0.0042$.
