# Supplementary material for: Longitudinal Correlation of Frequency-to-Place Mismatch and Postoperative Speech Perception Outcomes in Cochlear Implant Recipients: Monosyllable, Consonant, Word, and Sentence
Source: Audiol Res. 2026 Apr 10;16(2):56. doi: 10.3390/audiolres16020056 (PMC13114027; doi:10.3390/audiolres16020056)
Supplement: Supplementary file 1 [file audiolres-16-00056-s001.zip › Supplementary Table S2_AudiolRes.pdf]

Supplementary Table S2.
Partial correlations between angular insertion depth (AID) and speech perception outcomes

		3 month		6 month		12 month	
		Partial correlation coefficient	p-value	Partial correlation coefficient	p-value	Partial correlation coefficient	p-value
CI-2004	Monosyllable	0.23	0.201	0.38	0.035	0.44	0.030
	Consonant	0.42	0.012	0.47	0.008	0.54	0.005
	Word	0.27	0.123	0.25	0.154	0.32	0.121
	Sentence	0.30	0.091	0.24	0.191	0.28	0.157

Partial correlation analyses were performed, controlling for age and preoperative mean air-conduction thresholds (500, 1000, and 2000 Hz). Bonferroni-corrected significance threshold: $p < 0.0042$.
